# Fostering Age Inclusivity in Higher Education: Bridging Theory and Practice at the University of Haifa

**DOI:** 10.1093/geroni/igaf122.1935

**Published:** 2025-12-31

**Authors:** Malka Doron, Sigal Naim, Michal Elboim-Gabyzon, Tal Kohli-Hailovski, Stav Barouch, Orit Hirsch-Matsioulas, Anna Zisberg

**Affiliations:** The Center for Research and Study of Aging, University of Haifa, Israel; The Center for Research and Study of Aging, University of Haifa, Haifa, HaZafon, Israel; University of Haifa, Haifa, HaZafon, Israel; University of Haifa, Haifa, HaZafon, Israel; The Center for Research and Study of Aging, University of Haifa, Haifa, HaZafon, Israel; The Center for Research and Study of Aging, University of Haifa, Israel; The Center for Research and Study of Aging, University of Haifa, Haifa, HaZafon, Israel; University of Haifa, Haifa, HaZafon, Israel

## Abstract

The Age Inclusivity Domains of Higher Education (AIDHE) model guides universities in embedding age-friendly practices across seven key domains: Teaching and Learning, Services and Resources, Student Affairs, Physical Environment, Research, Personnel, and Outreach and Engagement. This presentation demonstrates how the University of Haifa has operationalized the AIDHE model since joining the global network of age-friendly universities last year through eight targeted, impactful initiatives, that align with the AIDHE’s seven domains: (1) a student volunteer project with older adults, fostering intergenerational connections and reducing old age related stereotypes; (2) faculty-led lectures for older adults, promoting lifelong learning and university-community collaboration; (3) designating parking spaces for older persons, enhancing campus accessibility; (4) promoting the transformation of the university into an age-friendly workplace; (5) partnering with the university’s sports center to develop a course certifying fitness trainers in older adult wellness; (6) collaborating with the city municipality to promote an age-friendly city; (7) developing an age-friendly university app that centralizes studies and activities for older adults, advancing digital inclusion; and (8) collaborating with the Ministry for Social Equality and Advancement of the Status of Women of Israel to integrate Government Resolution 127: ‘National Indicators Framework for Optimal Aging’, preparing for longer lifespans and supporting optimal aging. Some of these initiatives are accompanied by research involving students and the older adult community. The presentation provides a roadmap for adapting the model to the university’s unique academic, social, cultural, and environmental characteristics, highlighting the reciprocal benefits for students, faculty, staff, and the broader older community.

